# Bioactive Peptides from Andean Crops: Lactic Acid Bacteria Fermentation, Complementary Proteolysis, and Biological Activities

**DOI:** 10.3390/foods15162895

**Published:** 2026-08-19

**Authors:** Carlos Barba-Ostria, María José Barreno-Sánchez, Luis Fabián Salazar-Garcés, Jéssica Guamán-Bautista, Linda P. Guamán

**Affiliations:** 1 Escuela de Medicina, Colegio de Ciencias de la Salud Quito, Universidad San Francisco de Quito (USFQ), Quito 170901, Ecuador; 2 Instituto de Microbiología, Universidad San Francisco de Quito (USFQ), Quito 170901, Ecuador; 3 Carrera de Medicina, Facultad de Ciencias de la Salud, Universidad Técnica de Ambato, Ambato 180104, Ecuador; mj.barreno@uta.edu.ec (M.J.B.-S.); lf.salazar@uta.edu.ec (L.F.S.-G.); 4 Allergy and Acarology Laboratory, Institute of Health Sciences, Federal University of Bahia, Salvador 40110-100, Brazil; 5 Facultad de Ciencias de la Hospitalidad, Carrera de Gastronomía, Universidad de Cuenca, Cuenca 010201, Ecuador; jessica.guaman@ucuenca.edu.ec; 6 Centro de Investigación Biomédica (CENBIO), Facultad de Ciencias de la Salud Eugenio Espejo, Universidad UTE, Quito 170527, Ecuador

**Keywords:** bioactive peptides, lactic acid bacteria fermentation, quinoa, amaranth, chocho, cañihua, enzymatic hydrolysis, ACE inhibition, SDGs 2, and 3

## Abstract

Andean crops, including quinoa (*Chenopodium quinoa*), amaranth species including kiwicha (*Amaranthus caudatus*), chocho (*Lupinus mutabilis*), and cañihua (*Chenopodium pallidicaule*), constitute promising protein matrices for bioactive peptide generation by microbial fermentation and enzymatic proteolysis. These pseudocereals and legumes contain storage proteins, mainly 11S globulins and 2S albumins, enriched in lysine and sulfur-containing amino acids, and therefore provide suitable precursors for multifunctional peptides. In addition, secondary metabolites, such as quinoa saponins and chocho alkaloids, may influence proteolysis, peptide release, and peptide stability during fermentation. This review critically examines LAB fermentation as a food-grade strategy for bioactive peptide production from Andean crops. Evidence from enzymatic hydrolysis and simulated gastrointestinal digestion is included as complementary information and is discussed separately from fermentation-derived evidence. Reported activities include antioxidant, ACE-inhibitory, DPP-IV- and α-glucosidase-inhibitory, antimicrobial and anti-inflammatory activities. Particular attention is given to peptide generation, molecular-weight fractionation, peptidomic characterization, structure–activity relationships, simulated gastrointestinal digestion, and bioaccessibility. Most reported activities were measured in hydrolysates or molecular-weight fractions rather than in defined sequences, and individually validated peptides remain a minority. The strength of evidence differs markedly among crops: fermentation-derived data are more developed for quinoa and amaranth, whereas evidence for chocho and cañihua relies largely on enzymatic hydrolysis. Current limitations, including substrate variability, sensory acceptability, process standardization, peptide identification, bioavailability, and the lack of human validation studies, are also discussed. Andean crop proteins are promising precursors of functional peptide ingredients; however, peptide-specific bioavailability and human efficacy remain largely untested.

## 1. Introduction

Food-derived functional proteins and bioactive peptides are increasingly recognized as key components in the development of health-promoting food systems. Bioactive peptides are short amino acid sequences encrypted within dietary proteins that remain inactive until released through proteolytic processes such as enzymatic hydrolysis, gastrointestinal digestion, or microbial fermentation [1]. Once liberated, these sequences can act as molecular effectors that modulate physiological processes, including cardiovascular regulation, redox homeostasis, inflammatory responses, metabolic control, and host–microbe interactions [2,3,4]. Their multifunctional properties have positioned them at the interface of food science, nutrition, molecular biology, and functional food development [5]. In parallel, the transition toward sustainable and resilient food systems has intensified the search for plant-derived protein sources that extend beyond conventional nutritional value. Within this context, Andean crops have re-emerged as biochemically and agronomically relevant sources [6,7,8].

Crops such as quinoa, amaranth species, and chocho combine marked environmental adaptability with dense and structurally diverse protein matrices. When species-level evidence is available, kiwicha is treated as *Amaranthus caudatus*. Two features largely account for their potential as substrates for fermentation-driven bioactive peptide release. The first is their proteomic architecture. Andean crop proteins are mainly composed of 11S globulins and 2S albumins, which contain amino acid motifs enriched in lysine and sulfur-containing residues and serve as precursors of high-affinity bioactive sequences [9]. During fermentation, their distinct storage-protein composition may influence susceptibility to proteolysis and the resulting peptide profiles. However, direct comparative peptidomic evidence with conventional cereals remains limited [10,11]. The second feature is the presence of secondary metabolites. Phytochemicals such as quinoa saponins and chocho alkaloids are often regarded as anti-nutrients; however, in a biotechnological context, they may act as metabolic modulators that influence proteolytic efficiency and peptide stability during fermentation [12,13,14].

LAB fermentation is an important food-grade strategy for releasing bioactive peptides from Andean crop proteins and is the primary focus of this review [15,16]. However, fermentation-specific evidence is uneven among crops. Therefore, studies based on enzymatic hydrolysis or simulated gastrointestinal digestion are included when they provide complementary information on peptide sequences or activities, but they are explicitly identified as non-fermentative evidence.

Despite growing interest in plant-derived bioactive peptides, the evidence for Andean crops remains fragmented across different substrates, fermentation systems, peptide fractions, bioactivity assays, and mechanistic approaches. Many studies report antioxidant or ACE-inhibitory effects at the hydrolysate or fraction level [17,18,19], whereas fewer studies provide sequence-resolved peptide identification, molecular docking, simulated gastrointestinal digestion, bioaccessibility assessment, or in vivo validation [20,21,21,22]. Therefore, a critical synthesis is needed to clarify how LAB fermentation contributes to peptide generation, which biological activities are best supported, what mechanisms have been proposed, and which translational barriers still limit their application in functional foods and nutraceuticals.

This review critically evaluates bioactive peptides from Andean crops, with emphasis on those generated by LAB fermentation. Complementary evidence from enzymatic hydrolysis and gastrointestinal digestion is considered separately to identify bioactive sequences, compare functional activities, and define the current limitations of the field.

## 2. Proteomic Architecture and Secondary Metabolite Context of Andean Crops

**Figure 1:**
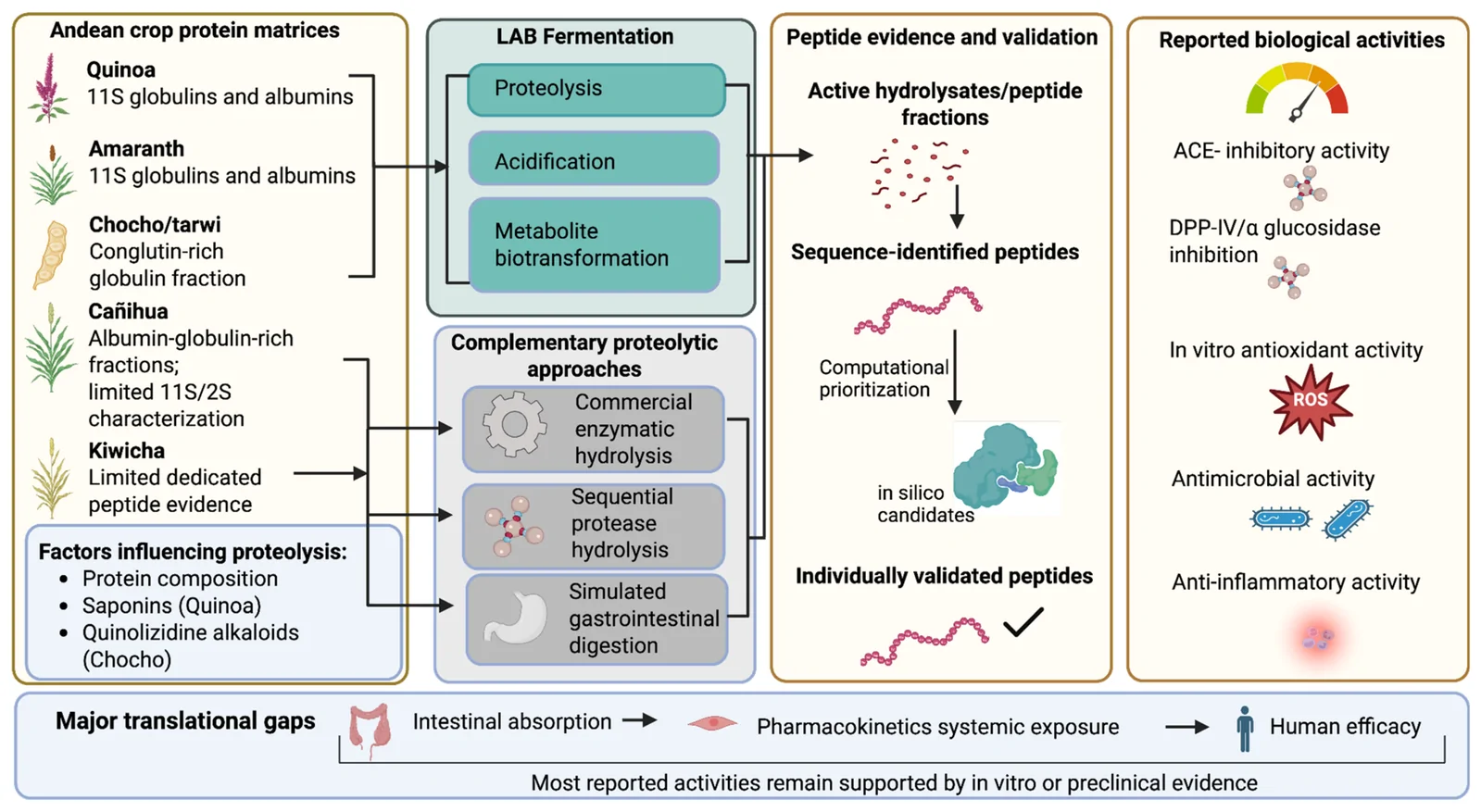
Evidence-based framework for bioactive peptide generation from Andean crop proteins. LAB fermentation represents the primary focus of this review, whereas enzymatic hydrolysis and simulated gastrointestinal digestion provide complementary evidence, particularly for crops with limited fermentation-specific studies. Solid arrows indicate direct experimental evidence and dashed arrows limited or inferential evidence. The framework distinguishes activity measured in hydrolysates or peptide fractions from sequence identification, individual peptide validation, and in silico prediction. Most reported bioactivities remain based on in vitro or preclinical evidence, whereas peptide-specific absorption, pharmacokinetics, and human efficacy remain poorly characterized.

Andean crops possess proteomic architectures that distinguish them from conventional cereals and make them suitable substrates for LAB fermentation [23]. Unlike cereal grains, which are dominated by prolamins with limited nutritional balance and compact structures that restrict proteolytic access, Andean storage proteins are composed mainly of albumins and globulins [24]. These fractions are enriched in 11S globulins and 2S albumins, scaffolds characterized by high levels of lysine, an essential amino acid often limited in cereals, and sulfur-containing residues such as cysteine and methionine [25]. The resulting amino acid motifs provide precursors for bioactive peptide sequences with affinity for molecular targets such as angiotensin-converting enzyme (ACE), dipeptidyl peptidase-IV (DPP-IV), and oxidative stress mediators. In addition, the relatively open tertiary and quaternary structures of these proteins, stabilized by inter- and intra-chain disulfide bonds but containing accessible hydrophilic domains, facilitate a diverse proteolytic peptide map under LAB-derived peptidases, yielding low-molecular-weight peptides (<3 kDa) with diverse bioactivities, as summarized in Figure 1.

In quinoa, mature seed protein consists of approximately 37% 11S-type globulin, known as chenopodin, and 35% 2S albumin, with minor fractions of glutelins and prolamins [26]. Chenopodin is a hexameric storage protein (~300–400 kDa) composed of acidic and basic subunits linked by disulfide bonds, whereas the smaller and more hydrophilic 2S albumins represent a distinct protein fraction. These structural differences are expected to influence proteolytic accessibility, although their specific contribution to peptide release during LAB fermentation has not been systematically quantified. Quinoa proteins also contain relatively high levels of lysine, methionine, and cysteine. Sulfur-containing residues may contribute to the antioxidant activity of selected peptides; however, peptide bioactivity ultimately depends on sequence context rather than amino acid composition alone [26,27]. These compositional traits reflect the evolutionary adaptation of *Chenopodium* species to high-altitude environments, resulting in storage proteins suited for rapid mobilization and nutritional efficiency.

Amaranth species display similar proteomic profiles, with 11S globulins, termed amarantin, representing the dominant storage fraction alongside 2S albumins and glutelins. Amarantin is a hexameric globulin of ~398 kDa under native conditions, composed of acidic (~32–34 kDa) and basic (~22–24 kDa) subunits, and is rich in essential amino acids, including lysine, leucine, and phenylalanine [28,29]. The 11S globulin fraction in amaranth accounts for 16–35% of total protein, whereas albumins represent 19–45%, producing a highly digestible matrix that contrasts with the prolamin-rich cereal proteome [30]. Structural studies indicate that amaranth 11S globulins contain hypervariable regions particularly amenable to enzymatic cleavage, enabling the generation of peptides during LAB fermentation. Kiwicha shows a comparable albumin–globulin composition and lysine-rich profile; however, dedicated fermentation studies remain too limited to infer comparable peptide yields or bioactivity [30,31].

Chocho is notable for its high total protein content, ranging from 38 to 53% on a dry-weight basis and reaching 41% in mature seeds, with globulins comprising 91–94% of the proteome. The predominant 11S-type globulins, including α-, β-, and δ-conglutins, share structural homology with soybean glycinin but show distinct amino acid compositions enriched in arginine and leucine, together with lysine levels that exceed those of many legumes [32]. Albumins represent only ~6%, making chocho a globulin-dominant platform whose proteins are highly susceptible to proteolysis and can release bioactive peptides with broad biological activities. Most sequence-resolved evidence currently derives from non-fermentative hydrolysis, and the peptide repertoire generated specifically by LAB remains poorly characterized [33]. The high biological value of chocho proteins, reflected in the marked improvement of protein efficiency ratio after methionine supplementation, further underscores their potential as precursors of bioactive fragments. The main proteomic features, key amino acids, protein fractions, and phytochemical or antinutrient components of selected Andean grains are summarized in Table 1.

The second feature supporting the use of Andean crops for peptide bioproduction is the presence of secondary metabolites that interact dynamically with the proteolytic environment during LAB fermentation. In quinoa, triterpenoid saponins, ranging from 0.22 to 15.04 mg/g across accessions and concentrated mainly in the pericarp, are traditionally considered anti-nutritional because of their bitterness and membrane-disrupting activity [41]. However, LAB strains such as *L. plantarum* and *L. paracasei* produce β-glucosidases and esterases that degrade saponins into less bitter aglycones and polysaccharides. LAB fermentation can reduce saponin content and alter matrix composition [42]. Fermentation-driven saponin reduction, often exceeding 90%, also increases antioxidant capacity by releasing bound phenolics and improving matrix digestibility.

In chocho, quinolizidine alkaloids, mainly lupanine together with sparteine, angustifoline, and minor congeners, may reach 3–5% of seed dry matter and exert bitter and protease-inhibitory effects in unprocessed seeds. Aqueous debittering or LAB fermentation reduces these alkaloids to trace residual levels, ≤0.002 g/100 g dry matter for lupanine and sparteine, below international safety thresholds of ≤0.2 g/kg [43]. Debittering and fermentation reduce quinolizidine alkaloid levels. However, their direct effect on peptide release or stability remains poorly defined.

Together, storage-protein composition and matrix phytochemicals define conditions that may influence proteolysis during fermentation. However, direct evidence demonstrating synergistic effects on peptide yield, diversity, or bioactivity remains limited. Likewise, standardized comparisons with conventional cereals are lacking [44,45,46]. These interactions are summarized in Figure 1.

## 3. Fermentation, Processing, and Peptidomic Characterization

Building on the biochemical context described above, LAB fermentation is an important food-grade strategy for releasing peptides from Andean crop proteins [47] and is the primary focus of this review. This process exploits the extracellular and intracellular peptidase systems of strains such as *L. plantarum*, *L. paracasei*, *L. rhamnosus*, and *E. faecium*, enabling controlled proteolysis under mild conditions [21,48,49]. Cell-envelope proteinases and intracellular peptidases act sequentially to hydrolyze albumins and globulins into low-molecular-weight peptides. This fermentation-driven peptide release pathway is depicted in Figure 1. Fermentation is commonly performed at 30–37 °C, with an initial pH of 6.0–7.0 and incubation times of 24–72 h [50,51]. During this period, lactic acid production lowers the pH, improving protein solubility and enzyme–substrate interactions while reducing anti-nutritional factors. Both solid-state fermentation (SSF) of whole flours or grains and submerged fermentation (SmF) of protein isolates have been used successfully. SSF requires less water and more closely resembles traditional Andean practices, whereas SmF provides greater process control and homogeneity. Comparative studies indicate that SSF with autochthonous *L. plantarum* on quinoa flour can double antioxidant capacity relative to unfermented controls, while SmF of quinoa protein with *L. paracasei* generates potent ACE-inhibitory peptides [48,52,53].

The standardized production workflow begins with the preparation of protein-rich substrates. Defatted flours or concentrates from quinoa, amaranth, chocho, or cañihua are subjected to aqueous or mild alkaline extraction, typically at pH 8–9 with NaOH, to recover the predominant albumin and globulin fractions [21,54,55]. The extracted proteins are then fermented under controlled solid-state or submerged conditions. Post-fermentation hydrolysates are clarified by centrifugation and fractionated by sequential ultrafiltration using membranes with defined molecular-weight cut-offs, commonly 10, 5, 3, and 1 kDa [54]. Several studies report enrichment of bioactivity in low-molecular-weight fractions, frequently below 3 kDa. However, molecular size alone does not determine activity, which also depends on peptide sequence, charge, hydrophobicity, and assay conditions. Moreover, enrichment in a low-molecular-weight fraction should not be interpreted as evidence of enhanced intestinal absorption unless transport is experimentally demonstrated [18,55,56]. MW-dependent enrichment of bioactivity is not unique to plant-derived hydrolysates. In chicken embryo protein hydrolysates, fractionation into 0.2 μm, 10 kDa, and 3 kDa fractions yielded distinct functional profiles: antioxidant and anti-inflammatory activities were broadly retained, whereas ACE-inhibitory activity was detected only in the 3 kDa fraction [57]. This comparison further indicates that molecular size can enrich specific activities, but does not uniformly predict bioactivity across endpoints or protein sources.

Peptidomic characterization of bioactive fractions is performed by liquid chromatography coupled to tandem mass spectrometry (LC-MS/MS). The workflow begins with peptide separation by reversed-phase high-performance liquid chromatography (RP-HPLC) or ultra-performance liquid chromatography (UPLC), followed by electrospray ionization (ESI) and collision-induced dissociation in the mass spectrometer [58,59]. Precursor ions and fragment spectra are acquired in data-dependent mode and matched against species-specific protein databases or interpreted using de novo sequencing algorithms. In quinoa, nano-LC-ESI-MS/MS has enabled the identification of 5–9-residue peptides from fermented flour extracts [22,60]. In amaranth fermented with *E. faecium* LR9, the same platform identified 125 novel sequences, of which 10 showed high predicted ACE-inhibitory potential (Table 2) [61,62]. Complementary secondary-structure analysis by circular dichroism (CD) spectroscopy indicates that many active peptides adopt α-helical or random-coil conformations that favor target binding [63].

**Table 2:** Experimental evidence for bioactive peptides and peptide fractions from selected Andean crops.

Crop	Processing Method	LAB Strain/Enzyme	Peptide or Fraction	Activity	Quantitative Evidence	Validation Status *	References
Quinoa	LAB fermentation + ultrafiltration/chromatographic purification	*Lactobacillus paracasei CICC 20241*	NIFRPFAPEL	ACE inhibition	IC_50_ = 49.02 μM	Individual peptide validated	[21]
Quinoa	LAB fermentation + ultrafiltration/chromatographic purification	*Lactobacillus paracasei CICC 20241*	AALEAPRILNL	ACE inhibition	IC_50_ = 79.72 μM	Individual peptide validated	[21]
Quinoa	Germination + LAB fermentation + fractionation	*L. casei*	VAHPVF	α-Glucosidase; ACE inhibition	Significant individual inhibition of α-glucosidase and ACE; peptide-specific IC_50_ not clearly reported.	Individual peptide tested	[50]
Quinoa	Germination + LAB fermentation + fractionation	*L. casei*	LAHMIVAGA	α-Glucosidase; ACE inhibition	Significant individual inhibition of α-glucosidase and ACE; peptide-specific IC_50_ not clearly reported.	Individual peptide tested	[50]
Quinoa	Mixed-strain fermentation + purification	Mixed bacterial culture	AGAAPE	Antibacterial	MIC = 5 mg/mL against *E. coli* and *S. aureus*	Individual peptide validated	[64]
Quinoa	Enzyme-assisted LAB fermentation + ultrafiltration	*Lactobacillus paracasei*; amylase + lipase pretreatment	0–3 kDa fraction	ACE inhibition; antibacterial activity against *E. coli*	Quantitative ACE IC_50_ not reported; 0–3 kDa fraction showed the highest/stable ACE-inhibitory activity	Active fraction/hydrolysate	[65]
Amaranth	LAB fermentation	*Enterococcus faecium* LR9	Fermented protein hydrolysate	ACE inhibition	79.1 ± 2.6% ACE inhibition; *L. mesenteroides* 18C6: 68.0 ± 9.8%; Alcalase: 69.4 ± 1.2%	Active fraction/hydrolysate	[66]
Amaranth	LAB fermentation + peptide identification/in silico screening	*E. faecium* LR9	IFQFPKTY	Predicted ACE-inhibitory activity	No experimental IC_50_ reported; prioritized by bioinformatics/molecular docking	In silico candidate	[66]
Amaranth	LAB fermentation + peptide identification/in silico screening	*E. faecium* LR9	VIKPPSRAW	Predicted ACE-inhibitory activity	No experimental IC_50_ reported; prioritized by bioinformatics/molecular docking	In silico candidate	[66]
Amaranth	Enzymatic protein hydrolysis + chromatographic purification	Proteolytic hydrolysis; no LAB	SSEDIKE	Anti-inflammatory/immunomodulatory	Reduced CCL20 expression in activated Caco-2 cells; oral synthetic peptide reduced IgE/IgG1 and IL-5/IL-13 responses in a mouse food-allergy model	Individual peptide validated	[67,68]
Tarwi/chocho (*L. mutabilis*)	Enzymatic hydrolysis + ultrafiltration/size-exclusion chromatography + peptide synthesis	Alcalase + Neutrase	AVPFWM	ACE inhibition; DPP-IV inhibition; antioxidant activity	ACE IC_50_ = 6.38 ± 3.45 μM; DPP-IV IC_50_ = 133.0 ± 33.2 μM; ABTS = 3.17 ± 0.13 μmol TE/μmol peptide; ORAC = 2.66 ± 0.19 μmol TE/μmol peptide	Individual peptide validated	[17,69]
Tarwi/chocho (*L. mutabilis*)	Enzymatic hydrolysis + peptide synthesis	Alcalase + Neutrase	YSGWLGL	ACE inhibition; DPP-IV inhibition; antioxidant activity	ACE IC_50_ = 0.14 ± 0.01 μM; DPP-IV IC_50_ = 16.4 ± 1.0 μM; ABTS = 3.94 ± 0.06 μmol TE/μmol peptide; ORAC = 3.10 ± 0.09 μmol TE/μmol peptide	Individual peptide validated	[69]
Tarwi/chocho (*L. mutabilis*)	Enzymatic hydrolysis + peptide synthesis	Alcalase + Neutrase	AHAGFGMLY	ACE inhibition; DPP-IV inhibition; antioxidant activity	ACE IC_50_ = 0.69 ± 0.09 μM; DPP-IV IC_50_ = 1140 ± 457 μM; ABTS = 2.12 ± 0.04 μmol TE/μmol peptide; ORAC = 1.64 ± 0.10 μmol TE/μmol peptide	Individual peptide validated	[69]
Tarwi/chocho (*L. mutabilis*)	Enzymatic hydrolysis + LC-MS/MS + in silico prediction	Alcalase + Neutrase	FFSMKVM	Predicted ACE- and DPP-IV-inhibitory activity	PeptideRanker score = 0.809; no individual experimental IC_50_ reported	In silico candidate	[17]
Tarwi/chocho (*L. mutabilis*)	Sequential simulated gastrointestinal hydrolysis	Pepsin + pancreatin	Hydrolyzed γ-conglutin (Cgh)	DPP-IV inhibition; glucose uptake; gluconeogenesis	100% DPP-IV inhibition at 5 mg/mL; 6.5-fold increase in glucose uptake; ~50% reduction in gluconeogenesis	Active fraction/hydrolysate	[70]
Tarwi/chocho (*L. mutabilis*)	Sequential gastrointestinal enzymatic hydrolysis + fractionation	Pepsin + pancreatin	RLGN, VNEGA, SEIGGA, SAPRST, GALGLGH, PQNLDL, AGGPQQR, PSELSGAAH, LPKHSDAD, LTFPGSAD	Predicted ACE-inhibitory activity; selected sequences also predicted as DPP-IV inhibitors	No individual experimental IC_50_ values reported	In silico candidate	[70]
Cañihua	Sequential enzymatic hydrolysis + ultrafiltration/size-exclusion chromatography	Neutrase + Alcalase	Fraction III (<3 kDa)	Antioxidant and ACE-inhibitory activity	Antioxidant activity = 3.18 μmol TE/mg; ACE inhibition = 78.4%; ACE IC_50_ = 55 μg/mL	Active fraction/hydrolysate	[39]
Cañihua	Sequential enzymatic hydrolysis + fractionation	Neutrase + Alcalase	LDKDYPKR	Associated with antioxidant and ACE-inhibitory active fractions	No individual activity reported; identified by LC-MS/MS in active fraction(s)	Sequence identified, not individually tested	[39]
Cañihua	Sequential enzymatic hydrolysis + fractionation	Neutrase + Alcalase	RLSAEKGVLYR	Associated with antioxidant and ACE-inhibitory active fractions	No individual activity reported; identified by LC-MS/MS in active fraction(s)	Sequence identified, not individually tested	[39]
Cañihua	Sequential enzymatic hydrolysis + fractionation	Neutrase + Alcalase	LFR	Associated with antioxidant and ACE-inhibitory active fractions	No individual activity reported; identified by LC-MS/MS in active fraction(s)	Sequence identified, not individually tested	[39]
Cañihua	Enzymatic hydrolysis + chromatographic purification	Alcalase or sequential pepsin–pancreatin digestion	Four purified antimicrobial peptide fractions	Antimicrobial activity against *E. coli*, *S. aureus*, and *C. albicans*	28/216 hydrolysates showed ≥45% growth inhibition; four active fractions were purified. A glutelin-derived fraction showed 52% inhibition of *S. aureus* and 70% inhibition of *C. albicans*; ~95% inhibition was reported for *E. coli*.	Active fraction/hydrolysate	[20]

Abbreviations: LC-MS/MS, liquid chromatography coupled to tandem mass spectrometry; ACE, Angiotensin-Converting Enzyme; DPP, Dipeptidyl Peptidase; MIC, Minimum inhibitory concentration; TE, Trolox equivalents; ABTS, (2,2′-azino-bis-3-ethylbenzothiazoline-6-sulfonic acid). * Validation status indicates whether bioactivity was demonstrated for an individual peptide, for a peptide-containing fraction or hydrolysate, or inferred from sequence-based prediction.

Enzymatic hydrolysis provides a complementary strategy for peptide generation. Because peptide profiles depend on substrate, protease specificity, LAB strain, degree of hydrolysis, and processing conditions, the available evidence does not support a general conclusion that either enzymatic hydrolysis or LAB fermentation is intrinsically superior [20,71]. Combined fermentation–hydrolysis strategies may further diversify peptide profiles, although direct comparisons with single-process approaches remain limited. Overall, the integrated LAB fermentation–fractionation–peptidomics pipeline generates bioactive peptides while improving sensory attributes, mineral bioavailability, and shelf-life through acidification and metabolite production. The combined fermentation–fractionation–peptidomics workflow provides a useful framework for peptide discovery, but reproducibility across crops and production scales still requires standardization.

## 4. Crop-Specific Evidence from Andean Food Matrices

Quinoa is the most extensively studied Andean crop for bioactive peptide generation through LAB fermentation. The solid-state fermentation of quinoa flour with autochthonous *L. plantarum* strains markedly increased DPPH radical-scavenging activity, reaching up to a 2-fold improvement over unfermented controls. This effect was associated with the proteolytic release of hydrophobic and aromatic amino acid-rich sequences and the concomitant biotransformation of bound phenolics [35]. The submerged fermentation of quinoa protein with *L. paracasei* CICC 20241 generated potent antihypertensive peptides, including NIFRPFAPEL, which inhibited ACE with an IC_50_ of 49.02 μM. This peptide acted competitively through hydrogen bonding with ACE active-site residues, including Tyr523 and Glu384, and retained substantial activity after simulated gastrointestinal digestion [34,72,73]. Fermented quinoa preparations have been associated with changes in gut microbial composition. However, these effects cannot presently be assigned specifically to fermentation-derived peptides, because viable LAB, organic acids, phenolics, residual carbohydrates, and other fermentation products may also contribute. Peptide-specific microbiome effects therefore remain unresolved [52,74,75].

Amaranth also provides direct evidence of peptide release during LAB fermentation. In one comparative study, *E. faecium* LR9 fermentation yielded 79.1 ± 2.6% ACE inhibition, compared with 68.0 ± 9.8% for *L. mesenteroides* 18C6 and 69.4 ± 1.2% after Alcalase treatment [18]. Complementary studies using mono- and co-cultures of *Lactobacillus casei* Shirota and *Streptococcus thermophilus* 54102 confirmed the release of peptides with antihypertensive, antithrombotic, and antioxidant activities from amaranth proteins [19]. Kiwicha (*Amaranthus caudatus*) has been studied mainly through enzymatic hydrolysis and simulated gastrointestinal digestion, not LAB fermentation [76,77,78]. Dedicated LAB fermentation studies on kiwicha remain limited.

Chocho (*L. mutabilis*) offers substantial, although still underexplored, potential for LAB fermentation-derived peptides. Current evidence comes mainly from alcalase-optimized enzymatic hydrolysis, which produced hydrolysates with a high degree of hydrolysis and strong antioxidant activity, with TEAC values of 2.7 μmol Trolox eq/mg and ORAC values of 3.8 μmol Trolox eq/mg, attributed to peptides enriched in hydrophobic and aromatic residues [79]. Ultrafiltration-derived peptide fractions from chocho protein hydrolysates also show ACE-inhibitory, DPP-IV-inhibitory, α-glucosidase-inhibitory, and antihypertensive properties. Several sequences, including RLGN, VNEGA, SEIGGA, SAPRST, GALGLGH, PQNLDL, AGGPQQR, PSELSGAAH, LPKHSDAD, and LTFPGSAD, have been identified as promising candidates for cardiovascular and antidiabetic applications [17]. Direct LAB fermentation studies on chocho remain scarce compared with pseudocereals. Nevertheless, these observations provide a rationale for testing whether selected LAB strains can combine debittering with peptide release in chocho.

Cañihua is the least-studied Andean pseudocereal in peptide-focused fermentation research. Enzymatic hydrolysis studies provide the main evidence of its bioactive potential. Sequential neutrase–alcalase treatment of cañihua protein concentrate for 180 min at 50 °C produced hydrolysates with strong antioxidant activity of 2.12 μmol TE/mg and ACE inhibition of 69.8%, with an IC_50_ of 0.12 mg/mL. Further ultrafiltration and size-exclusion chromatography yielded a <3 kDa fraction with 3.18 μmol TE/mg, 78.4% ACE inhibition, and an IC_50_ of 55 μg/mL. LC-MS/MS analysis identified 3–11 amino acid peptides derived from 11S seed globulin [39]. Additional alcalase and pepsin–pancreatin hydrolysates of cañihua glutelin fractions released antimicrobial peptides active against *Escherichia coli*, *Staphylococcus aureus*, and *Candida albicans*, with growth inhibition up to 95%, minimum inhibitory concentration (MIC) values in the low μg/mL range, anionic character, and degrees of hydrolysis reaching 67% [38]. These studies demonstrate that cañihua proteins contain bioactive peptide sequences. Whether LAB fermentation releases the same sequences or generates fractions with comparable activity remains unknown.

Across the crops examined, low-molecular-weight (MW) fractions are those most often associated with bioactivity, although this observation draws on both LAB fermentation and enzymatic hydrolysis studies [80]. Fermentation-derived evidence is confined largely to quinoa and amaranth. For chocho and cañihua, the record consists almost entirely of hydrolysis studies, which identifies a gap rather than providing a basis for extrapolation. The compositional factors underlying these crop-specific differences are summarized in Table 1.

## 5. Functional Properties and Bioactivities

Peptides and peptide-rich fractions from Andean crops show several in vitro bioactivities, although the evidence derives from both LAB fermentation and non-fermentative hydrolysis [39]. Across studies, the most active fractions are usually enriched in low-molecular-weight peptides, most often below 3 kDa. Low-molecular-weight fractions are frequently enriched in activity [17,18,66], but molecular size alone does not predict biological potency or intestinal absorption. However, the available evidence remains uneven across crops and bioactivities: antihypertensive and antioxidant effects are the best documented, whereas sequence-resolved antimicrobial and anti-inflammatory data are still comparatively limited, particularly for strictly LAB-fermented Andean matrices [20].

### 5.1. ACE-Inhibitory Activity

ACE inhibition is the most consistently quantified activity among Andean crop-derived peptides. In quinoa, solid-state fermentation with *L. paracasei* generated peptide-rich fractions in which the <3 kDa fraction showed high and stable ACE-inhibitory activity. NIFRPFAPEL and AALEAPRILNL were subsequently validated as individual ACE-inhibitory peptides, with IC_50_ values of 49.02 and 79.72 μM, respectively [21,21,22]. In a separate fermented quinoa system, sprouted quinoa yogurt beverages inoculated with *L. casei* strains yielded peptide fractions containing RGAIVL, GVRGRGRIV, GGRFA, LGGIWHL, VAHPVF, IRAMPVAV, ALFPTHR, and LAHMIVAGA. Two of these peptides, VAHPVF and LAHMIVAGA, were subsequently tested individually and showed ACE- and α-glucosidase-inhibitory activity. The IC_50_ values reported for the parent hydrolysates or fractions should not be assigned to these individual sequences. [50,65,81].

Fermentation of *A. hypochondriacus* with *E. faecium* LR9 yielded a hydrolysate with 79.1 ± 2.6% ACE inhibition. LC-MS/MS identified 125 peptide sequences, among which IFQFPKTY and VIKPPSRAW were prioritized by bioinformatic and docking analyses. Importantly, the quantitative ACE inhibition applies to the hydrolysate; neither peptide has been individually validated or assigned an experimental IC_50_ [66,82,83].

For chocho, data exist at the hydrolysate and at the individual-peptide level, but none of it comes from LAB fermentation. Enzymatic hydrolysis yielded a low-molecular-weight fraction containing 25 peptides, from which AVPFWM, YSGWLGL, AHAGFGMLY, and FFSMKVM were first selected in silico. Three of these were later synthesised and assayed, giving ACE IC_50_ values of 6.38 ± 3.45 μM for AVPFWM, 0.14 ± 0.01 μM for YSGWLGL and 0.69 ± 0.09 μM for AHAGFGMLY; FFSMKVM has not been tested [84]. Further chocho-derived sequences recovered after gastrointestinal digestion, including RLGN, VNEGA, SEIGGA, SAPRST, GALGLGH, PQNLDL, AGGPQQR, PSELSGAAH, LPKHSDAD, and LTFPGSAD, are supported only by sequence-based prediction [17,39,85].

The lowest ACE IC_50_ values compiled in this review therefore belong to synthesised peptides obtained after the enzymatic hydrolysis of chocho, not to fermentation-derived peptides, and whether LAB fermentation of the same substrate releases these sequences has not been examined. For cañihua, the evidence is likewise non-fermentative. In *C. pallidicaule*, sequential enzymatic hydrolysis followed by purification produced a fraction with antioxidant activity of 3.18 μmol TE/mg and ACE inhibition of 78.4%, with an IC_50_ of 55 μg/mL [39]. LC-MS/MS identified LDKDYPKR, RLSAEKGVLYR, and LFR within the active fractions; their individual contributions to ACE inhibition were not determined [34,40]. Cañihua can therefore be described as a source of ACE-inhibitory peptide fractions, but not yet of validated individual sequences (Table 2).

### 5.2. Antioxidant Activity

Antioxidant activity has been assessed using complementary assays, most commonly DPPH, ABTS, FRAP, ORAC, and metal-chelation methods, and generally increases after proteolysis and fractionation [78,86,87]. Fermented amaranth extracts showed increased ABTS and FRAP activity, although these assays do not establish the relative contribution of peptides versus other fermentation-derived metabolites [18].

In quinoa, fermentation and peptide enrichment also improve antioxidant performance, although many studies report activity at the fraction level rather than for individual sequences [86,88,89]. Fermentation with *L. plantarum* has been associated with increased DPPH scavenging activity, consistent with observations across fermented quinoa matrices [18,52]. Complementary evidence from quinoa peptide studies has identified sequences such as FHPFPR, NWFPLPR, and NIFRPF with reported antioxidant and ACE-inhibitory properties [33,90]. Although these peptides were not isolated from LAB-fermented systems, they further support the bioactive potential of quinoa-derived peptide fractions.

Tarwi offers stronger sequence resolution for antioxidant activity. The low-molecular-weight chocho fraction reported by Chirinos et al. yielded four prioritized peptides: AVPFWM, YSGWLGL, AHAGFGMLY, and FFSMKVM. AVPFWM, YSGWLGL, and AHAGFGMLY were subsequently synthesized and experimentally confirmed to display antioxidant activity, whereas FFSMKVM remains a computationally prioritized candidate [70]. Follow-up work on synthesized tarwi peptides reported ABTS antioxidant capacity between 2.12 and 3.94 μmol TE/μmol peptide and ORAC values between 1.64 and 3.10 μmol TE/μmol peptide [17,39].

Cañihua again provides clear quantitative evidence. Its purified low-molecular-weight fraction reached 3.18 μmol TE/mg, and LDKDYPKR, RLSAEKGVLYR, and LFR peptides were identified within the active fractions, but their individual antioxidant activities were not measured [39].

### 5.3. DPP-IV and α-Glucosidase Inhibition

DPP-IV and α-glucosidase inhibition are commonly used as in vitro endpoints to screen food-derived peptides for potential effects on postprandial glucose regulation. This assay quantifies their ability to inhibit dipeptidyl peptidase-IV activity, thereby limiting the proteolytic degradation of incretin hormones such as glucagon-like peptide-1 (GLP-1), a mechanism relevant to incretin-mediated glucose regulation [17,91,92]. In fermented quinoa beverages, two peptides identified after *L. casei* fermentation, VAHPVF and LAHMIVAGA, were reported as dual ACE and α-glucosidase inhibitors. However, peptide-specific IC_50_ values for these sequences should not be assigned unless directly reported in the primary study [51,65]. These findings support the further evaluation of fermented quinoa peptides, but their quantitative potency remains insufficiently resolved at the individual-sequence level. These findings suggest that fermentation may enhance the release of bioactive peptides with potential applications in glycemic control and cardiovascular health [50].

Additional quinoa fermentation studies also support enzyme inhibition at the fraction level [89,91,92]. Fermented sprouted quinoa yogurt beverages exhibited dual α-glucosidase and ACE inhibition, while solid-state fermentation enhanced α-glucosidase inhibition relative to unfermented controls [50,70,93]. However, most studies characterize bioactivity at the fraction level, with limited identification and quantitative evaluation of individual peptide sequences.

Tarwi/chocho-derived peptides have shown notable DPP-IV-inhibitory properties. Hydrolyzed γ-conglutin preparations from *L. mutabilis* strongly inhibited DPP-IV; in a 2018 cellular study, the highest dose of the hydrolyzed γ-conglutin fraction (Cgh, 5 mg/mL) produced inhibition comparable in magnitude to sitagliptin, while also enhancing glucose uptake and reducing gluconeogenesis by approximately 50% in a dual enterocyte/hepatocyte system [17,70,94]. Although the individual peptide sequences responsible for DPP-IV inhibition were not identified, this study provides one of the strongest mechanism-oriented antidiabetic references for an Andean crop.

The broader tarwi hydrolysate study by Chirinos et al. adds a sequence-level layer: 25 peptides were detected in the active low-molecular-weight fraction, and AVPFWM, YSGWLGL, AHAGFGMLY, and FFSMKVM were prioritized as candidates with predicted DPP-IV- and ACE-inhibitory potential [17]. These findings provide sequence-level candidates that may contribute to the DPP-IV- and ACE-inhibitory activity of tarwi hydrolysates [39,66], but they do not establish physiological effects in vivo.

Compared with quinoa and tarwi, evidence for antidiabetic peptides in amaranth and cañihua remains more limited [17,51]. Although amaranth protein hydrolysates have shown DPP-IV-inhibitory activity and improved glucose tolerance in animal models, only a small number of bioactive sequences have been characterized [95,96]. For cañihua, studies evaluating DPP-IV- or α-glucosidase-inhibitory peptides remain scarce. Further work is therefore needed to identify specific bioactive sequences, clarify their mechanisms of action, and validate their efficacy in vivo [17,97,98].

### 5.4. Antimicrobial Activity

In quinoa, antimicrobial evidence comes from both saponin fractions and peptide-rich hydrolysates. Antimicrobial activity is another relevant functional property of peptides derived from Andean crops, particularly because peptides, protein hydrolysates, and saponins can inhibit foodborne pathogens and spoilage microorganisms. Most studies have assessed antimicrobial activity using inhibition zone assays, minimum inhibitory concentration (MIC), minimum bactericidal concentration (MBC), and antifungal growth inhibition tests. These studies show that fermentation, enzymatic hydrolysis, and peptide purification can enhance bioactivity by releasing low-molecular-weight antimicrobial compounds [99,100].

In quinoa, both saponins and peptide-rich hydrolysates contribute to antimicrobial activity. Quinoa saponins inhibited a broad range of microorganisms, including *S. aureus*, *Staphylococcus epidermidis*, *Bacillus cereus*, *E. coli*, *Porphyromonas gingivalis*, *Clostridium perfringens*, and *Fusobacterium nucleatum* [100,101,102] Alkali-transformed quinoa saponins showed particularly strong antibacterial activity against *F. nucleatum*, with MIC and MBC values of 31.3 and 125 μg/mL, respectively [101]. Antifungal activity has also been reported against *C. albicans*, *Botrytis cinerea*, *Alternaria arborescens*, and *Phytophthora cinnamomi* [103,104,105,106]. In addition, quinoa protein hydrolysates produced inhibition zones of 11.88 mm against *Streptococcus pyogenes* and 12.49 mm against *E. coli*, confirming the antimicrobial potential of peptide-enriched fractions [64,107,108,109,110].

Amaranth also provides substantial antimicrobial evidence through both native proteins and peptide hydrolysates. Antimicrobial proteins containing cysteine/glycine-rich domains have shown activity against Gram-positive bacteria and several phytopathogenic fungi, suggesting a protective role in plant defense mechanisms [111]. Peptide fractions isolated from *A. hypochondriacus* and *Amaranthus retroflexus* inhibited the growth of *C. albicans*, *Fusarium solani*, *Fusarium oxysporum*, *Alternaria alternata*, and *Aspergillus ochraceus* [112,113]. Protein hydrolysates obtained by enzymatic digestion also inhibited *S. aureus*, *Salmonella typhimurium*, *E. coli*, and *Enterobacter aerogenes*, indicating that proteolysis enhances the release of antimicrobial peptides from amaranth proteins [109,110].

Tarwi shows more limited but promising antimicrobial evidence. Although specific antimicrobial peptide sequences have not been comprehensively characterized, the high protein content of *L. mutabilis* and the nature of its hydrolysates suggest potential antimicrobial applications. Current studies have focused mainly on antioxidant, antihypertensive, and antidiabetic activities, underscoring the need for further investigation of antimicrobial mechanisms and peptide identification in tarwi-derived fractions [17,84,99].

Cañihua contributes emerging evidence through peptide fractions obtained from enzymatically hydrolyzed seed proteins. Sequential alcalase and pepsin–pancreatin hydrolysis generated antimicrobial peptide fractions capable of inhibiting bacterial and fungal microorganisms, supporting the potential use of cañihua-derived peptides as natural antimicrobial agents [20]. Although quantitative antimicrobial parameters are less extensively characterized than in quinoa or amaranth, these findings indicate that cañihua proteins are a promising source of bioactive peptides for food preservation and functional food applications.

### 5.5. Anti-Inflammatory Activity

Anti-inflammatory effects have been linked to modulation of NF-κB and NLRP3 inflammasome signaling pathways. In quinoa, these effects have been associated with saponins, peptides, and protein hydrolysates. Notably, several anti-inflammatory observations in quinoa derive from saponin fractions or whole extracts rather than peptide preparations and therefore should not be interpreted as peptide-specific effects [106]. Similarly, quinoa-derived peptides downregulated NF-κB signaling and promoted PPAR-γ activation, contributing to reduced inflammatory responses in endothelial and intestinal cell models [90]. Quinoa protein hydrolysates also increased IL-10 production and modulated cytokine release, while young green quinoa extracts strongly inhibited TNF-α and IL-6, supporting their potential role in controlling chronic low-grade inflammation [114,115,116,117].

Amaranth has also shown anti-inflammatory potential through peptide-rich hydrolysates. Protein hydrolysates from *Amaranthus caudatus* reduced pro-inflammatory cytokine production and downregulated inflammasome-related genes while enhancing anti-inflammatory mediators in intestinal cell models [118]. In addition, peptides released during the gastrointestinal digestion of germinated amaranth showed antioxidant and anti-inflammatory activities, largely associated with the modulation of NF-κB signaling and the NLRP3 inflammasome pathway [67,119,120].

For chocho and cañihua, direct evidence remains limited. Although both crops contain high-quality proteins and bioactive compounds with antioxidant and metabolic benefits, studies specifically evaluating inflammatory biomarkers and molecular mechanisms are still scarce [17,84,87].

### 5.6. Methodological Heterogeneity and Interpretation of Evidence

Comparison among studies is limited by substantial methodological heterogeneity. Peptides have been generated by LAB fermentation, commercial proteases, sequential enzymatic hydrolysis, and simulated gastrointestinal digestion under markedly different conditions [121,122]. Substrate preparation, protease specificity, fermentation time, pH, temperature, and degree of hydrolysis all influence the resulting peptide profile [121,123]. Therefore, results obtained with different processing strategies should not be considered directly equivalent.

A second limitation concerns peptide resolution. Bioactivity is frequently measured in whole hydrolysates or molecular-weight fractions, whereas peptide sequences are subsequently identified by LC-MS/MS [124,125,126]. The identification of a peptide within an active fraction does not demonstrate that the peptide itself accounts for the measured activity [124,125]. Sequence-specific activity requires the isolation or synthesis of the peptide, followed by direct experimental testing [126].

Quantitative comparisons also require caution. Percentage inhibition measured at a single hydrolysate concentration cannot be directly compared with an IC_50_ determined for a purified peptide. Likewise, IC_50_ values expressed in μM for individual peptides are not directly comparable with values expressed in mg/mL for complex fractions [126]. Antioxidant assays such as DPPH, ABTS, FRAP, and ORAC measure related but distinct chemical properties and should not be treated as equivalent measures of biological efficacy [127,128,129].

Accordingly, Table 2 distinguishes four levels of peptide-specific evidence: individually validated peptides, active fractions or hydrolysates, identified but untested sequences, and in silico candidates. [130,131]. These categories should be distinguished from physiological validation, because in vitro activity does not establish gastrointestinal stability, absorption, systemic exposure, or efficacy in humans [132,133,134].

## 6. Molecular Mechanisms, Docking, and Structure–Activity Relationships

Molecular docking and structure–activity relationship (SAR) analyses have helped define how fermentation-derived peptides from Andean crops interact with their molecular targets [69,85]. Together with enzyme inhibition kinetics and cellular assays, these approaches identify binding modes, interacting residues, and structural features associated with bioactivity [135,136]. Docking supplies a structural hypothesis for a peptide–target interaction; it does not demonstrate inhibition [60]. Predictions are therefore treated here as supporting evidence only where experimental data are also available [131,137].

For ACE inhibition, docking studies show that Andean peptides bind within the catalytic site or adjacent regions through hydrogen bonds, hydrophobic contacts, and electrostatic interactions. The quinoa-derived peptide NIFRPFAPEL (IC_50_ = 49.02 μM) acts as a competitive inhibitor, forming hydrogen bonds with Tyr523 and Glu384, while its C-terminal leucine contributes hydrophobic anchoring within the ACE active site [138,139]. A second quinoa-derived peptide, AALEAPRILNL, shows non-competitive inhibition by interacting outside the catalytic pocket and stabilizing the enzyme–peptide complex without directly blocking substrate access [65,140]. In amaranth, IFQFPKTY and VIKPPSRAW, identified after *E. faecium* LR9 fermentation, show strong predicted binding to the ACE S1 and S2 subsites. Their aromatic and hydrophobic residues establish π–π stacking and van der Waals contacts with His383, His387, and Tyr523, yielding predicted affinities comparable to or higher than those of reference inhibitors [18,66]. Chocho peptide fractions also show high docking scores against ACE, with arginine- and leucine-rich sequences contributing additional electrostatic stabilization [99,141,142]. These interactions are consistent with the ACE-inhibitory potency reported for fermented Andean hydrolysates relative to enzymatic controls.

Docking studies of antidiabetic targets further illustrate the functional versatility of Andean peptides. Quinoa-derived low-molecular-weight fractions inhibit α-glucosidase through mixed-type reversible kinetics. Molecular simulations suggest that hydrophobic patches and proline residues induce conformational changes in the enzyme active site, reducing substrate affinity without fully occluding the catalytic nucleophile [66,140]. Chocho hydrolysates also dock to DPP-IV and α-glucosidase. Proline-containing sequences occupy the DPP-IV S1 pocket through hydrogen bonding with Glu205 and Tyr662, whereas hydrophobic residues strengthen α-glucosidase binding, in agreement with their bioactive profile observed in vitro [33,142].

SAR analyses across Andean peptide datasets reveal conserved features associated with bioactivity. ACE-inhibitory peptides are typically 2–12 residues long, with hydrophobic amino acids such as Leu, Ile, Val, and Phe at the C-terminus and aromatic or branched-chain residues at the N-terminus. These features are well represented in the protein scaffolds of Andean crops [17,142,143]. Antioxidant activity correlates with Tyr, Trp, Phe, Cys, Met, or His residues, which support electron or hydrogen donation. DPP-IV inhibition is favored by proline at the penultimate position, whereas net positive charge and amphipathicity contribute to antimicrobial membrane disruption [144]. Circular dichroism data indicate that many active sequences adopt α-helical or flexible random-coil conformations that facilitate induced-fit binding. Molecular dynamics simulations further suggest that these complexes remain stable under physiological pH and temperature, supporting their structural stability under simulated physiological conditions [99,140,143].

Together, docking and SAR data indicate that the proteolytic cleavage patterns of Andean 11S/2S globulins and 2S albumins, modulated by secondary-metabolite interactions during LAB fermentation, generate peptides with favorable binding features. These mechanistic insights help explain the observed bioactivities and support the design of peptide libraries for targeted cardiometabolic and anti-inflammatory applications [18,18,52].

## 7. Gastrointestinal Stability and Bioaccessibility

In addition to molecular interactions with target enzymes, gastrointestinal stability is a key determinant of the potential bioaccessibility and physiological efficacy of fermentation-derived peptides. In quinoa, the antihypertensive peptide NIFRPFAPEL, released during *L. paracasei* fermentation, shows high resistance to simulated gastrointestinal digestion [21,21]. Sequential exposure to pepsin during the gastric phase and pancreatin during the intestinal phase largely preserves peptide integrity, with only limited degradation, while ACE-inhibitory activity remains at levels compatible with potential antihypertensive effects [21]. Fermented quinoa matrices also show that post-digestion hydrolysates may exhibit increased antioxidant capacity, likely due to the generation of secondary bioactive fragments during transit. These observations have been reported in INFOGEST-compliant static digestion models and in dynamic systems that more closely reproduce physiological conditions [145].

Amaranth fermented hydrolysates also retain substantial bioactivity after digestion. Dynamic in vitro digestion of amaranth-based beverages using the DIDGI^®^ system showed that ACE-inhibitory capacity was preserved in both gastric (IC_50_ = 80 ± 10 μg mL^−1^) and intestinal phases (IC_50_ = 140 ± 20 μg mL^−1^) [120]. Purification of the final digest yielded even more active fractions, with an IC_50_ of 60 ± 10 μg mL^−1^ in the most potent subfraction, confirming that bioactive peptides can survive gastrointestinal transit and become further enriched [18,145]. Encapsulation strategies, such as alginate/pectin beads, further improve retention of ACE-inhibitory activity in amaranth hydrolysates during simulated digestion, highlighting the relevance of formulation approaches for enhancing bioaccessibility [19].

Kiwicha protein hydrolysates subjected to simulated gastrointestinal digestion release bioactive peptides with high bioaccessibility. Digestibility reaches approximately 71–79% after 240 min of in vitro digestion, with the intestinal phase promoting the release of essential amino acids and bioactive fragments [145]. Fractions >5 kDa show pronounced effects on colon cancer cell viability, whereas <5 kDa fractions predominate in ACE inhibition and antioxidant activity, indicating that gastrointestinal processing can generate or enrich bioactive species [145].

Data for chocho and cañihua remain more limited, particularly for fermentation-derived peptides. Enzymatic hydrolysates of chocho proteins show good in vitro digestibility and retain antioxidant, ACE-inhibitory, and antidiabetic activities after digestion, whereas cañihua hydrolysates generate low-molecular-weight peptides that remain active after simulated gastrointestinal exposure [93,146]. Their storage proteins resemble those of quinoa and amaranth, but no fermentation–digestion study has been performed on either crop, so their behaviour after LAB fermentation is unknown [147].

Recent standardized digestion studies using the INFOGEST 2.0 protocol provide additional context on the gastrointestinal behavior of Andean crop proteins, although most have examined non-fermented matrices. In quinoa and cañihua, INFOGEST 2.0 digestion revealed high protein digestibility (71–79%) and the release of bioactive fragments with retained antioxidant and ACE-inhibitory activity [145]. Similar dynamic digestion experiments on amaranth-based beverages, calibrated to INFOGEST 2.0, showed preservation of ACE-inhibitory peptides in both gastric and intestinal phases, with further enrichment of active fractions after purification [148]. These findings also reveal an important gap: INFOGEST 2.0 has not yet been applied to LAB-fermented Andean matrices, limiting direct comparison with the fermentation-derived peptides discussed in this review.

The fractions below 3 kDa that predominate after LAB fermentation of Andean crops show reasonable stability during simulated digestion [119]. Some sequences resist complete proteolysis and, in a few reports, give rise to additional active fragments during intestinal transit [149,150]. These observations concern bioaccessibility rather than bioavailability. Surviving digestion does not establish that a peptide crosses the intestinal epithelium or reaches the circulation in an active form [151,152]. After luminal digestion, peptides encounter the mucus layer, the epithelial barrier, and brush-border and intracellular peptidases. Di- and tripeptides are transported by PepT1, whereas longer sequences are thought to cross by paracellular diffusion, transcytosis, or transporter-independent routes, and transport efficiency varies strongly with sequence [150,152]. For Andean crop-derived peptides, no study has yet combined a standardised digestion model with an epithelial transport system and targeted LC-MS/MS detection of the transported sequences, which is the minimum required to move from bioaccessibility to bioavailability [151].

## 8. Translational Challenges and Future Perspectives

Preclinical evidence exists for select peptides and hydrolysates, but translation into commercial products remains limited. Most studies have been performed at laboratory or small-pilot scale, under conditions that do not fully reflect the operational and economic constraints of industrial production. One major limitation is germplasm standardization, which affects peptide profiles and bioactivity reproducibility. Quinoa accessions differ widely in saponin content, ranging from 0.22 to 15.04 mg/g dry weight, with sweet varieties containing <0.11% and bitter varieties exceeding 1% [34,153]. These compounds can interfere with protease activity and protein hydrolysis kinetics, thereby altering peptide release [154]. Chocho also presents substantial variability in quinolizidine alkaloids, with levels from 0.5% to 3–5%, and up to 10% in raw seeds. These alkaloids impart intense bitterness and may inhibit LAB, compromising fermentation performance [37,155]. Amaranth varieties likewise differ in protein content (13–18%) and amino acid composition, influencing the availability of encrypted bioactive sequences [71,120]. Agronomic factors, including soil composition, altitude, water stress, and harvest timing, further increase batch-to-batch variation, complicating the standardization of raw materials and the quality-control systems required for industrial production and regulatory compliance [95,156,157,158].

Industrial fermentation must therefore balance several objectives: effective debittering of chocho alkaloids (≤0.002 g/100 g), controlled degradation of quinoa saponins without off-flavor formation, maximization of bioactive peptide yield, and maintenance of microbial safety [37,155]. The choice between solid-state fermentation (SSF) and submerged fermentation (SmF) is central to this process. SSF can yield higher peptide concentrations and better resemble traditional Andean processing, but scale-up is hindered by heterogeneous temperature and pH gradients and limited process control [159,160,161]. SmF offers better control, sterility, and scalability in established bioreactor systems, although it requires higher water inputs and additional downstream concentration steps [47,162]. In both systems, LAB strain selection remains critical. Suitable strains must combine efficient proteolytic activity with tolerance to inhibitory compounds such as saponins and alkaloids [73]. However, strain-dependent differences in enzymatic activity and tolerance still limit standardization and reproducibility [163,164,165,166].

Sensory acceptability, particularly bitterness, is another major barrier to consumer acceptance and commercial viability. Bitterness arises mainly from hydrophobic amino acids, including leucine, isoleucine, valine, phenylalanine, tyrosine, and tryptophan, exposed during proteolysis. This effect is especially relevant in peptides of 1–3 kDa, the same range often associated with optimal bioactivity [35,90,167]. Traditional debittering strategies, including adsorption and extraction, can reduce bioactivity because they remove peptides non-selectively [82]. Encapsulation approaches, such as alginate–pectin beads, chitosan-based systems, and liposomal carriers, can mask bitterness while preserving functional properties, including ACE-inhibitory activity, during simulated gastrointestinal digestion [83,168,169]. Nevertheless, these technologies increase production costs and require further validation under industrial processing and storage conditions, particularly regarding stability under thermal and mechanical stress [170,171].

Bioavailability remains a critical translational challenge. Although some low-molecular-weight peptides show resistance to gastrointestinal digestion and may undergo intestinal transport through carrier-mediated or paracellular pathways, gastrointestinal stability alone does not guarantee biological efficacy [172,173]. Following digestion, bioactive peptides must cross the intestinal epithelium, survive first-pass metabolism in enterocytes and the liver, enter the systemic circulation, reach their molecular targets, and remain sufficiently stable to exert physiological effects [152,174]. The efficiency of intestinal absorption depends on peptide size, amino acid sequence, hydrophobicity, charge, and susceptibility to brush-border peptidases [174]. While di- and tripeptides are efficiently transported by PepT1, larger oligopeptides are thought to cross the intestinal barrier through limited paracellular diffusion, transcytosis, or other transporter-independent mechanisms, although these pathways remain incompletely characterized [150]. Consequently, many peptides displaying potent in vitro bioactivities may undergo extensive degradation before systemic absorption, whereas others generate smaller fragments that retain or even enhance biological activity [149,150,174].

Most Andean crop-derived peptides have not been comprehensively evaluated using advanced in vitro models, such as Caco-2 monolayers, or in vivo systems [132,175]. Existing studies report variable outcomes: some peptides retain bioactivity after digestion, whereas others are rapidly degraded [138,176]. Importantly, virtually no human pharmacokinetic data are currently available for fermentation-derived peptides from quinoa, amaranth, chocho, or cañihua. Consequently, key pharmacokinetic parameters including absorption efficiency, plasma bioavailability, metabolic transformation, tissue distribution, elimination, plasma half-life, and dose–response relationships remain largely unknown [177,178]. This lack of ADME (absorption, distribution, metabolism, and excretion) information represents one of the principal barriers to translating promising in vitro findings into clinically relevant functional foods or nutraceuticals [152,179]. Future studies should therefore combine standardized digestion models with intestinal transport assays, LC-MS/MS-based pharmacokinetic analyses, and well-designed animal and human intervention studies to determine systemic peptide exposure, identify circulating bioactive metabolites, and establish clinically meaningful dosing regimens [151]. Integrating these experimental approaches with in silico ADME prediction and peptide engineering may further facilitate the rational design of fermentation-derived peptides with improved oral bioavailability and therapeutic potential [132,179]. Together with other barriers, these limitations define the research priorities summarized in Table 3.

Regulatory frameworks further complicate commercialization. In the European Union, health claims require authorization based on strong evidence from well-designed randomized controlled trials demonstrating a clear cause–effect relationship [180,181]. In the United States, structure–function claims are permitted but must be scientifically substantiated, whereas disease-related claims require full regulatory approval [180,182]. At present, the evidence base for Andean crop peptides relies mainly on in vitro assays and animal models, with a marked lack of robust human clinical trials. This gap limits regulatory approval, market acceptance, and the development of standardized functional ingredients [46,183,184].

Four requirements follow from the gaps identified above: standardised processing and bioactivity assays, validation of candidate sequences as isolated or synthesised peptides, measurement of intestinal transport and pharmacokinetics, and controlled studies in humans with endpoints such as blood pressure, glycaemic control, inflammatory markers, and gut microbiota composition, using standardised preparations from defined crop accessions [45,185]. Strain engineering, including CRISPR-based approaches, may produce LAB with higher proteolytic activity and greater tolerance to saponins and alkaloids [44,166]. In parallel, multi-omics approaches integrating peptidomics, metabolomics, and metagenomics can provide a systems-level understanding of fermentation and peptide generation, facilitating rational process optimization [160,186]. Computational tools, including molecular docking and machine learning, can help rank candidates before experimental work [69,85]. These methods complement experimental validation rather than substituting for it; the limiting factor at present is not the number of predicted candidates but the number that have been tested [96,99].

Finally, the development of bioactive peptides from quinoa, amaranth, and chocho aligns with global priorities for sustainable food systems. These crops are well adapted to adverse environmental conditions, including drought, salinity, and poor soils, making them valuable resources for agricultural diversification and food security [6,95]. Addressing the current translational bottlenecks through collaboration among food scientists, biotechnologists, clinicians, and regulatory experts will be essential to convert the biochemical potential of Andean crops into clinically validated, economically viable, and sustainable functional foods and nutraceuticals.

Table 3 summarizes the main limitations, scientific rationale, and future perspectives for the translation of fermentation-derived bioactive peptides from Andean crops.

**Table 3:** Translational challenges and research priorities for Andean crop-derived bioactive peptides.

Domain/Principle	Scientific Basis	Translational Challenges	Future Perspectives/Research Frontiers	Key References
Raw Material Variability	Andean crops contain diverse protein matrices with latent bioactive peptides	High variability in saponins (0.22–15.04 mg/g), alkaloids (0.5–10%), protein composition → poor reproducibility	Standardized germplasm; genomic-assisted crop selection; controlled agro-processing systems	[73,153]
Antinutritional Compounds	Saponins and alkaloids influence enzymatic hydrolysis and microbial growth	Inhibition of LAB and proteases; altered peptide release kinetics	Coupled detoxification–fermentation systems; tolerant or engineered LAB strains	[37,73,155]
LAB Proteolysis	LAB release peptides via proteolytic systems (CEP + peptidases)	Strain-dependent variability; lack of reproducibility and control	CRISPR-engineered LAB; designer starter cultures; synthetic consortia	[37,44,155,166]
Fermentation Systems (SSF vs. SmF)	SSF mimics traditional systems; SmF allows industrial control	SSF: scaling issues, gradients; SmF: dilution, cost, downstream burden	Hybrid fermentation; smart/automated bioreactors	[159,160]
Process Optimization Trade-offs	Need to balance peptide yield, detoxification, and safety	Conflicting targets: debittering (≤0.002 g/100 g), saponin removal, microbial safety	AI-driven multi-objective optimization; digital twins	[37,155]
Peptide Multifunctionality	Selected peptides and peptide fractions show multiple in vitro bioactivities	Hard to validate multi-target effects in vivo; unclear structure–function relationships	Systems biology; network pharmacology; multitarget nutraceutical design	[35,163,164]
Sensory Constraints (Bitterness)	Hydrophobic and sequence-dependent peptide features can contribute to bitterness	Debittering reduces bioactivity; limits consumer acceptance	Selective enzymatic hydrolysis; smart encapsulation strategies	[82,90,167]
Encapsulation & Delivery	Protects peptides and masks taste	High cost; instability under heat/mechanical stress; scale-up issues	Nanoencapsulation; co-delivery systems; stimuli-responsive carriers	[83,168,171]
Bioavailability	Selected small peptides may cross the intestinal epithelium through carrier-mediated, paracellular, endocytic, or passive pathways	Peptide-specific transport, systemic exposure, and human pharmacokinetics remain largely uncharacterized	Organoid models; human pharmacokinetics; peptide engineering	[172,173,175]
Clinical Validation Gap	Preclinical evidence exists for selected peptides and hydrolysates	Human efficacy, dose–response relationships, and clinically relevant exposure remain largely unknown	Precision nutrition trials; biomarker-based interventions	[168,178]
Industrial Scale-Up	Fermentation is scalable but complex	High costs, purification challenges, batch variability	Process intensification; membrane filtration; green extraction	[37,180]
Gut Microbiome Interactions	Fermented Andean matrices have been associated with microbiome changes	Peptide-specific causality is unresolved because LAB, fibre, phenolics, organic acids, and other fermentation products may contribute	Purified-peptide studies and controlled fermented-matrix comparisons	[45,185]
Computational Prediction	In silico tools predict peptide bioactivity	Limited integration with experimental workflows	AI/ML peptide discovery; molecular docking pipelines	[69,85]
Sustainability & Crop Valorization	Andean crops are climate-resilient and nutrient-dense	Underutilized supply chains; limited industrial adoption	Sustainable biorefineries; global market integration	[6,95]
Functional Food Integration	Peptides incorporated into food systems	Matrix interactions reduce activity; sensory issues	Clean-label functional foods; plant-based innovations	[182,183]
Mechanistic Understanding	Mechanisms are well defined for some enzyme-inhibitory peptides but remain uncertain for many cellular or systemic effects	Limited mechanistic clarity in humans	Integrated in vitro–in vivo–in silico validation	[132,175,177,185]

Abbreviations: CEP, Cell Envelope Proteinase; CRISPR, Clustered Regularly Interspaced Short Palindromic Repeats; AI, Artificial Intelligence; ML, Machine Learning; SSF, solid-state fermentation; SmF, submerged fermentation.

## 9. Conclusions

Current evidence supports Andean crop proteins as precursors of bioactive peptides, although the strength and origin of that evidence differ markedly among crops. Direct LAB-fermentation evidence is best developed for quinoa and amaranth, whereas studies on chocho and cañihua rely predominantly on enzymatic hydrolysis or simulated gastrointestinal digestion.

ACE inhibition and antioxidant activity are the most consistently reported endpoints. However, much of the evidence remains at the hydrolysate or peptide-fraction level. The identification of a sequence within an active fraction does not establish sequence-specific activity, and molecular docking should be regarded as predictive rather than experimental evidence.

Translation remains limited by methodological heterogeneity, incomplete peptide validation, uncertain intestinal absorption and systemic exposure, and the near absence of human pharmacokinetic and intervention studies. Likewise, microbiome effects reported for fermented matrices cannot yet be attributed specifically to peptides.

Future work should therefore prioritize standardized processing and bioactivity assays, the direct validation of candidate peptides, intestinal transport and pharmacokinetic studies, and controlled human trials. These steps are required before the biochemical activities of Andean crop-derived peptides can be translated into evidence-based functional food claims.

## Figures and Tables

**Table 1:** Comparative compositional features of selected Andean crops relevant to peptide generation.

Crop	Protein Content (% Dry Weight)	Major Storage Protein Fractions	Key Amino Acid Features	Major Phytochemicals/Antinutritional Factors	References
Quinoa (*C. quinoa* Willd.)	12.9–16.5	11S globulin (chenopodin) ≈ 37% 2S albumin ≈ 35%	Relatively high sulfur-amino-acid content compared with many cereals; however, methionine + cysteine may remain the limiting amino acids relative to FAO/WHO reference amino-acid patterns in some datasets	Triterpenoid saponins present at concentrations ranging from 0.22 to 15.04 mg/g, predominantly located in the pericarp	[21,34,35,36]
Amaranth (*A. hypochondriacus* L. and *A*. *caudatus* L.)	13–19	11S globulin (amarantin) 16–35% Albumins 19–45%	Lysine content reported between 5.0 and 6.0 g/100 g protein in multiple studies; sulfur-containing amino acid content generally higher than in most cereals	Triterpenoid saponin content generally lower than in quinoa	[18,19]
Chocho/Tarwi (*L. mutabilis* Sweet)	32.0–52.6	Globulins ≈ 91–94% (predominantly 11S-type conglutins α, β, δ) Albumins ≈ 6%	Lysine content generally adequate relative to FAO/WHO patterns; methionine frequently identified as the limiting amino acid	High content of quinolizidine alkaloids (primarily lupanine and sparteine) in raw seeds	[33,37]
Cañihua (*C. pallidicaule* Aellen)	15–19 (up to 20% in selected accessions)	Albumin- and globulin-rich fractions; specific 11S/2S distribution remains incompletely characterized	Lysine content reported around 5.0–5.8 g/100 g protein in available studies.	Triterpenoid saponin content generally lower than in quinoa	[20,38,39,40]

Abbreviations: FAO, Food and Agriculture Organization; WHO, World Health Organization. Cañihua (*C. pallidicaule*), the least-studied Andean pseudocereal, shares several quinoa-like proteomic traits. Its protein content ranges from 15 to 19%, reaching up to 20% in selected accessions, and its albumin–globulin profile is enriched in lysine and sulfur-containing amino acids [31]. Although specific 11S/2S quantification is less documented than in quinoa, compositional analyses confirm a predominance of water- and salt-soluble fractions, resembling cleavage-accessible scaffolds of related *Chenopodium* species [38]. This similarity provides a rationale for testing cañihua as a fermentation substrate, although direct evidence remains limited (Table 1).

## Data Availability

No new data were created or analyzed in this study. Data sharing is not applicable.
